# Experimentally induced incomplete burst fractures - a novel technique for calf and human specimens

**DOI:** 10.1186/1471-2474-13-45

**Published:** 2012-03-25

**Authors:** René Hartensuer, Adam Gasch, Dominic Gehweiler, Steffen Schanz, Martin Schulze, Lars Matuszewski, Martin Langer, Michael J Raschke, Thomas Vordemvenne

**Affiliations:** 1 Department of Trauma-, Hand-, and Reconstructive Surgery, Westfälische Wilhelms-University Münster, Waldeyerstrasse 1, Münster 48149, Germany; 2 Department of Clinical Radiology, Westfälische Wilhelms-University Münster, Albert-Schweitzer-Straße 33, Münster 48149, Germany

**Keywords:** Incomplete burst fractures, Magerl A3.1, Calf, Human, Spine, Experimental fracture induction

## Abstract

**Background:**

Fracture morphology is crucial for the clinical decision-making process preceding spinal fracture treatment. The presented experimental approach was designed in order to ensure reproducibility of induced fracture morphology.

**Results:**

The presented method resulted in fracture morphology, found in clinical classification systems like the Magerl classification. In the calf spine samples, 70% displayed incomplete burst fractures corresponding to type A3.1 and A3.2 fractures. In all human samples, superior incomplete burst fractures (Magerl A3.1) were identified by an independent radiologist and spine surgeon.

**Conclusions:**

The presented set up enables the first experimental means to reliably model and study distinct incomplete burst fracture patterns in an *in vitro *setting. Thus, we envisage this protocol to facilitate further studies on spine fracture treatment of incomplete burst fractures.

## Background

The treatment of incomplete burst fractures is one of the most controversially discussed issues in spinal traumatology.

To our knowledge, neither clinical trials nor *in vitro *approaches have been able to reveal an exhaustive understanding of the pathology of this fracture type and its corresponding treatment needs to date.

In the absence of clear evidence-based recommendations on how to treat this type of injury, a whole range of surgical and nonsurgical options can be found in literature [1].

Holdsworth initially introduced the definition of "burst fractures" in 1970 [2], which was primarily considered to be a stable fracture. In contrast, clinical studies suggested [3,4], and experimental studies by Panjabi et al. [5] and Kifune et al. [6] revealed the instability of burst fractures. They observed that injuries to the middle column (according to the 3 column theory from Denis [3]) corresponded best with increased instability.

However, there is a discrepancy between the clear biomechanical estimation of instability and the controversial discussion in clinical treatment [1]. One possible reason could be the ambiguous definition of burst fractures. To compare biomechanical results, it seems to be mandatory that fracture morphology is rated by using classification systems, which are established in clinical routine.

**Figure 1:**
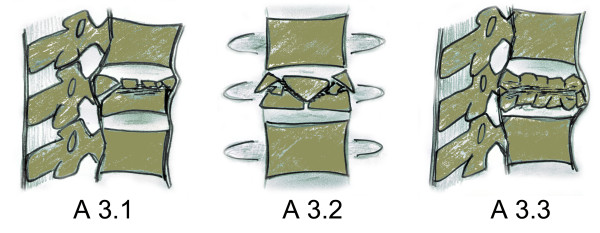
**Diagram of Magerl's subclassification of burst fractures A3.1 to A3.3**.

Magerl et al. published a reliable classification system in 1994 [7] which provides an excellent distinction between different subtypes of burst fractures. It has since become a common tool in clinical care and is popularly known as the AO classification. According to Magerl et al., compression type fractures are summarized as Type A fractures, Type B injuries are described by compression-distraction mechanism and Type C injuries include all rotational injuries. Burst fractures represent a subgroup of Type A injuries (A3) and are subclassified into incomplete burst fractures (A3.1), burst-split fractures (A3.2) and complete burst fractures (A3.3). The posterior ligamentous complex (PLC) remains intact in all Type A injuries (Figure 1).

Multiple techniques to inflict experimental burst fractures have been described to date [5,6,8-13]. A modified technique used by Kifune et al. [6] and Panjabi et al. [5] on human spine samples increases the dropping mass until a fracture occurs, which usually resulted in multiple attempts to produce the desired fracture. Using this approach, a wide range of fracture types from simple endplate fractures, wedge fractures to burst fractures can be inflicted.

Shono et al. used an impact load apparatus to create L1 burst fractures based on a hydraulic material testing device [12].

However, a method capable of producing finer grained morphologies of burst fractures *in vitro *corresponding to commonly use clinical fracture classifications, such as the AO classification is still lacking.

There is still an incomplete understanding of mechanical and neurological stability of this injury. Consecutive there are no clear commonly accepted treatment algorithms. Treatment options vary from conservative to invasive dorso-ventral treatment. On this account, the lack of an experimental means to produce appropriate subtypes of burst fractures is a pressing issue.

### Hypothesis

The presented protocol provides a method to reliably induce incomplete burst fractures in calf and human spine samples that correspond to existing fracture classification systems.

## Results

### Calf spine specimens

**Figure 2:**
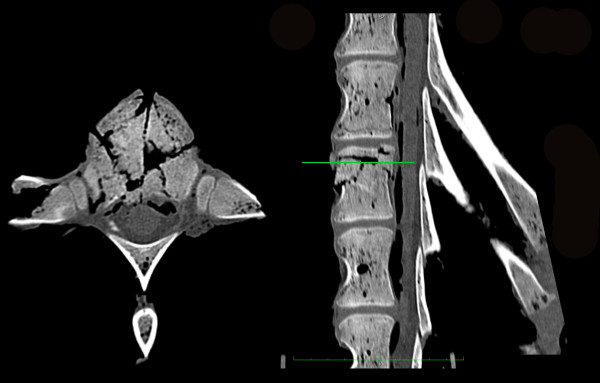
**CT scans showing example of the produced incomplete burst fracture in calf spine samples**.

In the calf spine samples, 70% displayed incomplete burst fractures corresponding to type A3.1 (40%) and A3.2 fractures (30%; Table 1), providing evidence that incomplete burst fractures can be reproduced with our protocol (Figure 2). The remaining fractures were rated as compression fractures (A1.2). The growth plate was involved in all fracture patterns. However, as described above, all osteotomy-like lesions cut through the caudal aspect of the vertebral disc, the endplate, the physis and the cranial aspect of the vertebral body. So fracture propagation following the growth plates resulted in further fragmentation of the produced fracture of the cranial vertebral body.

**Table 1:** Overview of calf specimens used for fracture production and consecutive radiological rating according to the Magerl/AO classification

	Target vertebral body	Compression (mm)	Force (kN)	Magerl/AO
1	Th13	8	14489	A3.1
2	L2	8	10356	A1.2
3	Th10	10	6523	A3.2
4	L2	10	6880	A3.2
5	Th13	10	6880	A3.2
6	Th9	10	7389	A1.2
7	Th13	10	8223	A1.2
8	L4	10	12732	A3.1
9	L1	10	9711	A3.1
10	L1	10	6615	A3.1

Thus, to produce incomplete burst fractures, only one single compression was sufficient using the presented protocol. A distance-controlled compression resulted in the fracture of the desired morphology.

The resulting forces to the target vertebra ranged between 7 to 14 kN (Table 1).

### Human spine samples

In all human samples, a fracture resulted in the target vertebral body by performing only a single compression cycle. An average failure load of 3.6 ± 1.3 kN was recorded.

**Figure 3:**
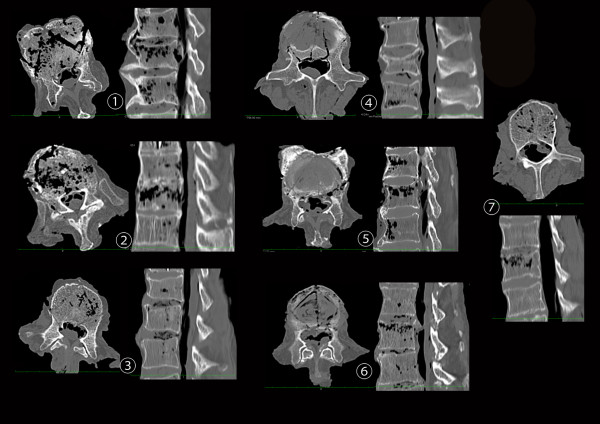
**CT scans showing representative axial and sagittal slices of the produced incomplete burst fracture in human spine specimens**.

In all samples (100%), superior incomplete burst fractures (Magerl A3.1.1) were identified (Figure 3).

The load sharing classification ranged from 4 to 7 points with an average rating of 5 ± 1.15. Evaluations via CT scan and macroscopic inspection of the specimens showed no signs of injury to the facets or posterior ligamentous complex (PLC) or rotational injury.

Thus, to produce incomplete burst fractures, a compression of approximately 20% of the vertebral body height resulted in the desirable fracture morphology in human spine samples using the described set up (Table 2).

**Table 2:** Overview of human samples used for fracture production and consecutive radiological rating according to the Magerl/AO and Load-sharing classification

	Target vertebral body	Compression (mm)	Force (kN)	Magerl/AO classification	Load Sharing Classification	Specimen	BMD (mg Ca-HA/ml)	T-Score	Age	Sex
1	Th 12	10.24	3.926	A 3.1.1	6	WS 75/08	62.2	-4.25	80	male
2	Th 12	10.26	5.358	A 3.1.1	7	WS 80/08	143	-0.59	88	female
3	L 1	10.26	2.673	A 3.1.1	4	WS 71/08	111.1	-2.4	75	male
4	L1	10.27	3.276	A 3.1.1	5	WS 110/08	103	-2.71	73	male
5	L1	10.25	5.333	A 3.1.1	5	WS 89	40.7	-4.3	89	female
6	L 1	10.26	2.941	A 3.1.1	4	WS 74/08	79.5	-2.89	87	female
7	L 1	8.21	1.886	A 3.1.1	4	WS 77/08	84.7	-2.7	75	female

## Discussion

The presented method provides a means to produce paradigmatic incomplete burst fracture patterns in calf and human 4-FSU samples, which simulate injuries in human patients seen regularly in clinical practice. To our knowledge, this is the first technique capable of reproducibly induce incomplete burst fractures.

The presented protocol tries to simulate the described mechanism of Type A injuries of the thoracolumbar junction [7]. According to Magerl's work, injuries are caused by axial compression of the spine with or without flexion with an almost exclusive effect on the vertebral body.

The presented procedure has taken advantage of the published classical approaches to study burst fractures, which utilized spine fragments, mounted onto a fracture apparatus. The apparatus induces fracturing by dropping a mass element on the spine specimen or high speed vertical compression by a hydraulic material testing apparatus [12].

In addition, a distance-controlled mode of compression, which defines the impaction depth and velocity, was developed. Utilising this model, the used force is adapted to each specimen's resistance.

The presented protocol is limited by the required structural damage (temporary plate/screw fixation) to produce the desired fracture type in calf and human specimen. No relevant additional damage caused by the inserted screws after compression load was observed. However, this might be a limitation for further investigations.

### Calf spine samples

Calf spines are commonly used specimens for biomechanical spine testing. Based on their biomechanical properties including motion range, calf spines are considered to be suitable specimens for implant systems and surgical procedures [14]. Other studies have used calf vertebrae for investigating implant characteristics [15,16]. However, important differences compared to human spines have been reported [14,17,18]. Thus, several distinct features of calf spines should be taken into account.

In spite of anatomical similarities, Cotteril et al. [17] found a greater length of the bovine spinous processes at distinct thoracic levels, and a greater length of transverse lumbar processes at L3 compared to human spines. These features might influence motion properties of calf spines. In addition, ligaments and muscle forces may play an important role [17]. Especially in multi-segmental testing, Riley et al. reported significant differences in axial rotation and lateral bending [19].

In addition, the metabolic parameters of calf vs. human spines warrant a critical view.

Swartz et al. found calf spines to be a suitable model for testing surgical implants and demonstrated that the values of equivalent mineral density (EMD) of 6- to 8-week old calf vertebrae match the density reported for young adult human vertebrae [18]. However, no comparative data on endplate strength or cortical bone property of calf vs. human spine specimens is available. In our study, we used spines of 3- to 6-month old calves and no EMD or BMD values of the used calf spine samples have been recorded.

The age at which the calf spine appears to match the situation in adult human spine best is described as 6 to 8 weeks [14]. A limitation in this study is the use of 3- to 6-month old calves due to restrictions on the availability. Further, the plating used for fracture production might weaken the adjacent vertebral bodies. This needs to be considered by using distinct implants in the future.

The presence of the physis and anatomical differences in immature bovine samples compared to human spines may also have influenced fracture induction. However, as long as the availability of human spine specimens is a limiting factor in conducting similar experiments, the bovine model is a helpful tool in spite of the discussed limitations and considerations.

Thus, being aware of these considerations, our protocol provides the possibility for interesting future work using calf spine samples in this incomplete burst fracture model.

### Human postmortem samples

The objective of this study was not the evaluation of the required force to break a human or an immature bovine vertebra but the development of a reproducible method for further investigations. Thus, performing osteotomy-like lesions and distance-controlled compression were combined to modify classical protocols.

Kifune et al. [6] revealed that up to 4.8 kN (57 Nm) is needed to break the human endplate. The recorded average failure load of 3.6 ± 1.3 kN in this study may be due to the utilised osteotomy-like endplate weakening or to possible differences in bone quality.

In contrast to most published protocols, five-segmental specimens have been used in this study. This may also have influenced the force required to induce the fracture.

Some authors have used a repeating dropping mass technique, a method that requires repeating the mass impact [5,16,20]. Using our modified method, only a single compression event is necessary to generate the fracture.

Shono et al. used a high-speed vertical compression to inflict L1 burst fractures in multi-segmental specimens. Therefore, the L1 vertebra and adjacent discs have been isolated by upper and lower box-shaped fixtures. Compression was performed under displacement control in a compressive direction until the distance between the upper and lower fixture was reduced to 10% of the original height in 0.5 seconds [12].

The axial compression of 20% of the original height of the target vertebra necessary in our protocol may have been due to a possible difference of rigidity of the used temporary fixation of the adjacent vertebrae.

However, the presented data imply that the use of a distance-controlled compression protocol provides excellent control in producing different fracture morphologies.

The same impact depth will be performed automatically even in spine specimens with more or less resistance so that the impact is automatically adapted to the used specimen. As indicated, ideal samples to study incomplete burst fractures would have been young human tissue. However, the presented technique resulted in incomplete burst fractures in osteoporotic human and young calf spine samples. This suggests that the presented technique might work on everything in-between and differences in bone quality may less influence the induction of similar injuries for biomechanical testing.

In our human samples, only minor differences in fracture morphology could be observed in specimens with different bone quality; thereby all fractures were rated as Magerl A3.1 fractures and a load sharing classification rating from 4 to 7 by an independent consultant radiologist and a senior spine surgeon.

## Conclusion

To our knowledge, this is the first approach to inflict incomplete burst fractures even in osteoporotic multi-segmental spine samples for further biomechanical investigation. The presented results indicate that induction of incomplete burst fractures in human and immature calf spine specimens are feasible.

The possibility of reproducible induction of distinct fracture types may provide a platform to conduct future studies into several aspects of clinically important treatment strategies of incomplete burst fractures. Thus investigations of spinal trauma care in an *in vitro *biomechanical set up to study the treatment of incomplete burst fractures can be facilitated.

## Methods

### Calf spine specimens

Ten fresh bovine spines aged between 3- to 6-month were obtained from a local abattoir (Westfleisch, Hamm, Germany). Specimens exhibiting signs of damage inflicted by the slaughter procedure were excluded from the study. The specimens were frozen after dissection and preparation. In all specimens used in this study, ligaments and joints were intact. Specimens were then thawed for 12 hours at room temperature; muscles and tendons were carefully dissected. All experiments were performed on five-vertebra segments of the thoraco-lumbar junction and lumbar region. The middle vertebra (3rd vertebral body) that was the target vertebral body for fracture creation of each specimen is presented (Table 1). The caudal and cranial vertebrae of the specimen were embedded in polymethylmethacrylate resin (PMMA; Technovit 3040, Heraeus Kulzer, Wehrheim, Germany) and left for 20 min to solidify. Subsequently, lesions of the target vertebra were performed as described below.

Five-vertebra segments were chosen to establish a model reliable to investigate different biomechanical effects in the treatment of incomplete burst fractures and to facilitate multi-segmental testing on the fractured samples.

### Human spine samples

Seven spinal segments consisting of 5 vertebrae were harvested from post-mortem donors of our anatomical institute and immediately frozen. All specimens were taken from the thoracolumbar junction. The average age of the specimens was 81 ± 6.9 years, with nearly equal sex distribution (m:f = 3:4). The local ethics committee approved the usage of post mortem samples of the local anatomical institution.

In all samples, bone mineral density (BMD) was measured using quantitative computed tomography (Q-CT) [21]. The average BMD was 89.17 ± 33.6 mg Ca-HA/ml and the average T-score of -2.83 ± 1.25 was calculated. Thus, except for one sample, only osteoporotic or osteopenic spine samples were available.

Just before testing, all specimens were thawed to room temperature. All soft tissue and muscles were dissected carefully to preserve the osseous and ligamentous structures. All samples were kept moist during the dissection and testing process.

The caudal and cranial vertebrae of the specimen were similarly prepared as the calf specimens. Subsequently, lesions of the target vertebra were performed as described below.

Despite the predominantly occurrence of burst fractures in younger population the presented study was performed in osteoporotic spine samples due to the availability of human post mortem tissue.

### Fracture induction using a servo-hydraulic device

To ensure that the fracture occurs to the target vertebra, a standardised osteotomy to the caudal endplate (details described below) was performed.

**Figure 4:**
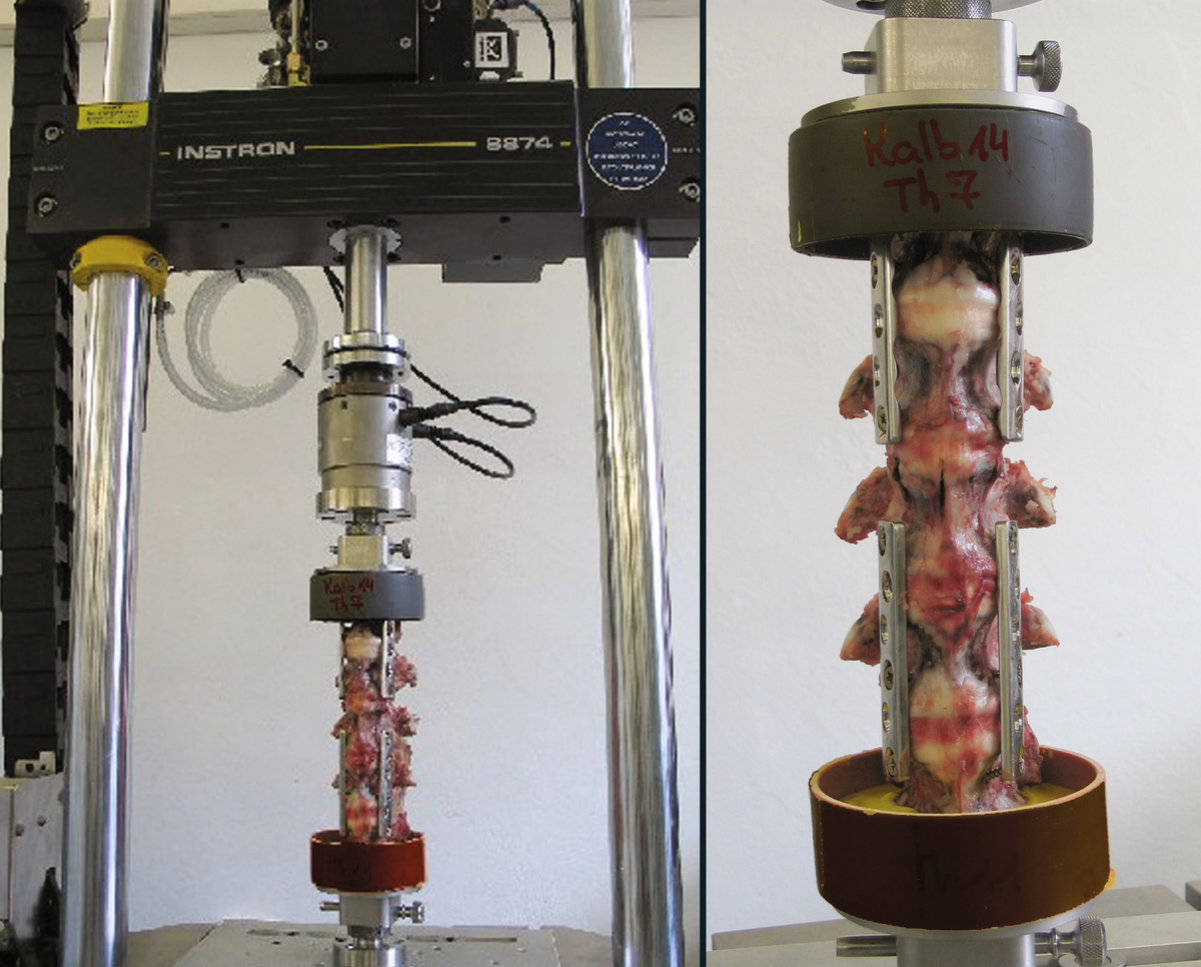
**Experimental setup, calf specimen mounted in the Instron servohydraulic material testing machine**.

Vertebrae above and underneath the intended break point were temporarily fused with dynamic compression steel plates (DCP, 4.5 mm) and screws. Therefore, only the upper half of the target vertebra and the adjacent cranial disc remained exposed between the upper and lower plate fixtures. The specimen was firmly mounted onto the Instron servo-hydraulic material testing device (Instron 8874, Instron Structural Testing Systems GmbH, Germany) in a 10° flexion angle (Figure 4). The specimen was then axially compressed under displacement control with a speed of 300 mm/s until the vertical distance was reduced to 20% of the original target vertebral body height.

The use of this set up enabled the reliable induction of incomplete burst fractures by a single compression event.

In order to minimise inertia effects on force measurements, a dynamic load cell (Dynacell, Instron Structural Testing Systems GmbH, Germany) was incorporated in the compression apparatus. Each specimen was checked macroscopically for signs of rotational or ligamentous injuries.

The maximum failure load was recorded for each specimen (Table 1). However, the specific aim of this study was to produce the fracture morphology comparable to incomplete burst fractures classified as Margerl A3.1.

### Osteotomy-like lesions of target vertebrae and temporary plating

Intact calf spine specimens used in this study appeared robust enough to tolerate force application within the limits of the force transducer of the compression device, which precluded fracture induction. Hence, a combination of compression and standardised weakening was developed. To this end, an osteotomy-like procedure to the cranial endplate of the target vertebra was performed using a surgical chisel (15-mm blade).

**Figure 5:**
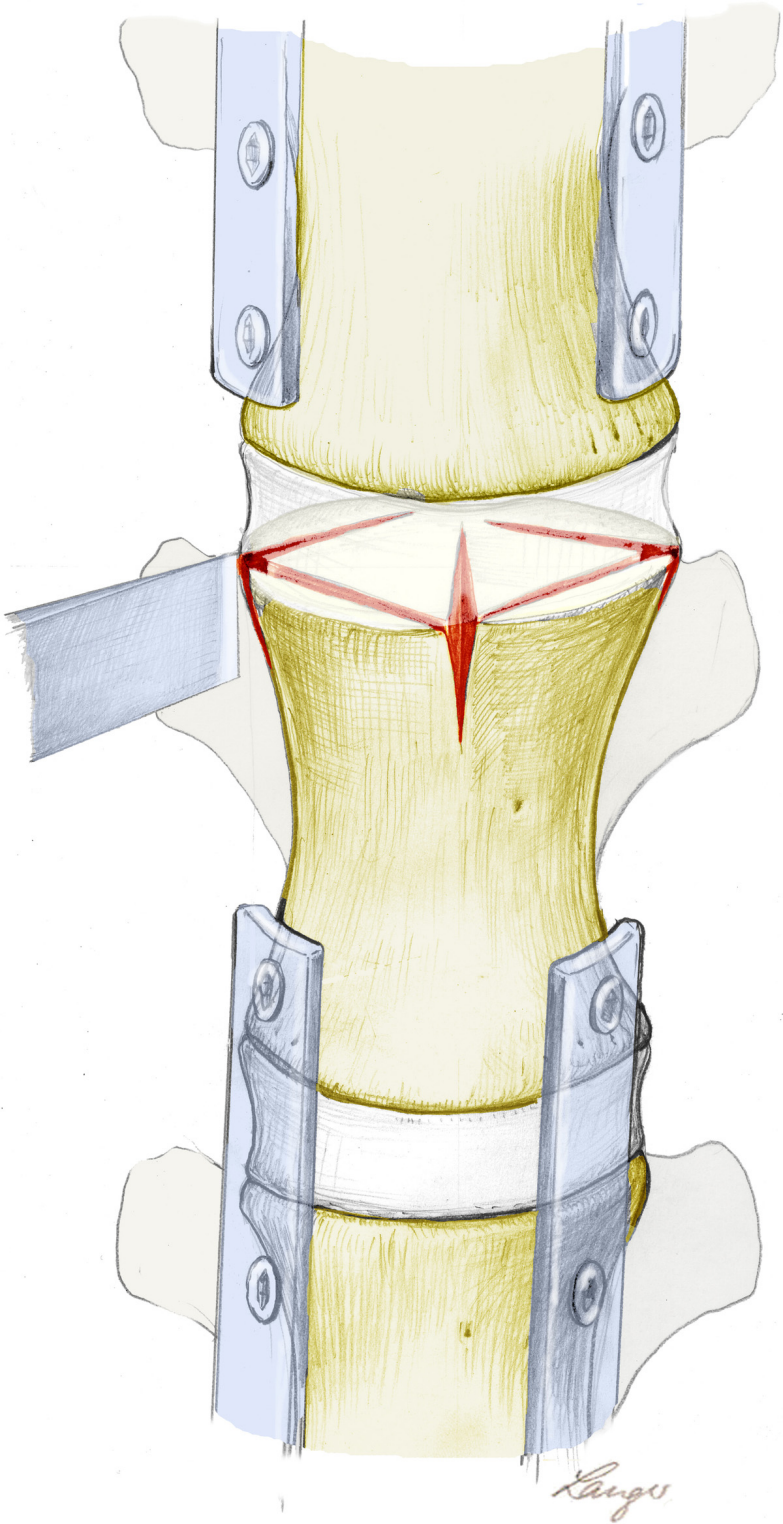
**Schematic position of Osteotomy-like lesions of target vertebrae and temporary plating**.

For all inflicted lesions in the calf specimens, it was ensured that the tool cut through the caudal aspect of the vertebral disc, endplate, physis and the cranial aspect of the vertebral body. The first lesion was applied in a straight anterior-posterior direction leaving the posterior wall of the vertebra intact. Subsequently, 2 oblique lesions from the anterior to the lateral-posterior aspect were inflicted. Finally, 2 converging lesions from the lateral side towards the posterior wall were applied leaving the posterior wall intact. In total, all lesions formed a rhomboid-shaped appearance (Figure 5).

This set up was then extrapolated to the human samples. In all specimens, the cranial endplate and the adjacent vertebral disc were injured using the described rhomboid-shaped technique.

### Radiological evaluation of fracture type

Computer-assisted tomography (CT) of spine specimens was conducted (Siemens Somatom Sensation 40). The CT scans had a slice thickness of 1.5 mm and a tube voltage of 120 kV, 34 mAs. Scan stacks were used to reconstruct 3-dimensional models of fractured vertebrae using the CT software package (Siemens Somaris Syngo 5). A consultant radiologist and a senior spine surgeon not involved in fracture induction experiments blindly scored the fracture types based on CT scans and classified the fractures according to the Magerl/AO classification. An additional rating according to the load sharing classification [22] was performed in human samples.

## Competing interests

The authors declare that they have no competing interests.

## Authors' contributions

RH conceived and planned the presented study. He observed and participated all presented biomechanical studies and drafted the manuscript. AG carried out the biomechanical studies and radiological investigation on calf spines. DG carried out the biomechanical studies and radiological investigation on human spines. SS planned and observed the technical implementation on bovine samples. MS observed and technically supported implementation on human samples. LM performed the radiological evaluation of the created fracture pattern. ML participated in drafting the manuscript and performed the presented schematic figure. MR participated in the design of the study and coordination. TV conceived of the study, and participated in its design and coordination and helped to draft the manuscript. All authors read and approved the final manuscript.

## Pre-publication history

The pre-publication history for this paper can be accessed here:

http://www.biomedcentral.com/1471-2474/13/45/prepub
